# Acute kidney injury subphenotypes based on creatinine trajectory identifies patients at increased risk of death

**DOI:** 10.1186/s13054-016-1546-4

**Published:** 2016-11-17

**Authors:** Pavan K. Bhatraju, Paramita Mukherjee, Cassianne Robinson-Cohen, Grant E. O’Keefe, Angela J. Frank, Jason D. Christie, Nuala J. Meyer, Kathleen D. Liu, Michael A. Matthay, Carolyn S. Calfee, David C. Christiani, Jonathan Himmelfarb, Mark M. Wurfel

**Affiliations:** 1 Division of Pulmonary and Critical Care Medicine, University of Washington, Harborview Medical Center, 325 9th Avenue, Seattle, WA 98104 USA; 2 Kidney Research Institute, University of Washington, Seattle, WA USA; 3 Division of Nephrology, University of Washington, Seattle, WA USA; 4 Department of Surgery, University of Washington, Seattle, WA USA; 5 Department of Environmental Health, Harvard T.H. Chan School of Public Health, Boston, MA USA; 6 Department of Epidemiology, Harvard T.H. Chan School of Public Health, Boston, MA USA; 7 Division of Pulmonary and Critical Care Division, Department of Medicine, Massachusetts General Hospital/Harvard Medical School, Boston, MA USA; 8 Division of Pulmonary, Allergy, and Critical Care, Perelman School of Medicine, University of Pennsylvania, Philadelphia, PA USA; 9 Center for Clinical Epidemiology and Biostatistics, Perelman School of Medicine, University of Pennsylvania, Philadelphia, PA USA; 10 Department of Medicine, University of California, San Francisco, CA USA; 11 Department of Anesthesia and Perioperative Care, University of California, San Francisco, CA USA; 12 Cardiovascular Research Institute, University of California, San Francisco, CA USA

**Keywords:** Critical care, Acute kidney injury, Mortality, Subphenotypes, Trajectory, Intensive care unit

## Abstract

**Background:**

Acute kidney injury (AKI) is common among intensive care unit (ICU) patients. AKI is highly heterogeneous, with variable links to poor outcomes. Current approaches to classify AKI severity and identify patients at highest risk for poor outcomes focus on the maximum change in serum creatinine (SCr) values. However, these scores are hampered by the need for a reliable baseline SCr value and the absence of a component differentiating transient from persistent rises in SCr. We hypothesized that identification of resolving or nonresolving AKI subphenotypes based on the early trajectory of SCr values in the ICU would better differentiate patients at risk of hospital mortality.

**Methods:**

We performed a secondary analysis of two prospective studies of ICU patients admitted to a trauma ICU (group 1; *n* = 1914) or general medical-surgical ICUs (group 2; *n* = 1867). In group 1, we tested definitions for resolving and nonresolving AKI subphenotypes and selected the definitions resulting in subphenotypes with the greatest separation in risk of death relative to non-AKI controls. We applied this definition to group 2 and tested whether the subphenotypes were independently associated with hospital mortality after adjustment for AKI severity.

**Results:**

AKI occurred in 46% and 69% of patients in groups 1 and 2, respectively. In group 1, a resolving AKI subphenotype (defined as a decrease in SCr of 0.3 mg/dl or 25% from maximum in the first 72 h of study enrollment) was associated with a low risk of death. A nonresolving AKI subphenotype (defined as all AKI cases not meeting the “resolving” definition) was associated with a high risk of death. In group 2, the resolving AKI subphenotype was not associated with increased mortality (relative risk [RR] 0.86, 95% CI 0.63–1.17), whereas the nonresolving AKI subphenotype was associated with higher mortality (RR 1.68, 95% CI 1.15–2.44) even after adjustment for AKI severity stage.

**Conclusions:**

The trajectory of SCr levels identifies AKI subphenotypes with different risks for death, even among AKI cases of similar severity. These AKI subphenotypes might better define the patients at risk for poor outcomes who might benefit from novel interventions.

**Electronic supplementary material:**

The online version of this article (doi:10.1186/s13054-016-1546-4) contains supplementary material, which is available to authorized users.

## Background

Acute kidney injury (AKI) is a heterogeneous syndrome defined by the Kidney Disease: Improving Global Outcomes (KDIGO) group as an increase in serum creatinine (SCr) of ≥0.3 mg/dl or >50% from baseline. The KDIGO group classifies patients from stage 0 (no AKI) to stage 3 AKI, based on maximum change in SCr or minimum urine output throughout the hospital stay. This definition for AKI includes a broad range of underlying pathophysiologic processes that would be expected to have different risks for poor clinical outcomes and may need to be treated differently. For instance, the KDIGO AKI definition does not differentiate between rises in creatinine due to temporary hemodynamic changes (e.g., volume depletion) versus true parenchymal injury (e.g., acute tubular necrosis). Even after classification by KDIGO severity stage, there is likely to be considerable clinical and biological heterogeneity. These limitations of the current AKI definition hamper the ability to better understand the pathophysiology of AKI and, potentially, the identification of effective novel therapies [1].

In clinical syndromes such as cancer, acute respiratory distress syndrome (ARDS), chronic obstructive pulmonary disease (COPD), and asthma, identification of subphenotypes has led to insights into their pathogenesis and the development of personalized approaches to care [2–8]. For example, Calfee et al. showed in patients with ARDS that indirect or direct lung injury is characterized by a unique biomarker pattern indicative of endothelial or epithelial dysfunction, respectively, suggestive of differences in the underlying pathobiology leading to these two forms of ARDS [5]. Additionally, in studies of asthma and COPD, researchers have employed a broad panel of clinical factors to identify subphenotypes [6, 8]. In our present study, we used creatinine kinetics to identify subphenotypes within AKI.

The trajectory of renal dysfunction is a potentially important and clinically intuitive parameter by which to understand AKI. Classifying AKI on the basis of trajectory rather than maximal creatinine change gets around the requirement for a preadmission “baseline” creatinine, which often is lacking for patients admitted to the intensive care unit (ICU) [9]. Classification based on trajectory also takes into account a patient’s response to early medical interventions and uses the added information provided by serial measures of renal dysfunction. Thus, identification of AKI subphenotypes based on the trajectory of SCr might improve the precision of risk stratification and provide more homogeneous groups of AKI cases.

We hypothesized that classifying patients with AKI into resolving and nonresolving subphenotypes on the basis of the trajectory of changes in SCr within the first 72 h of enrollment would result in groups with low and high associations with death, respectively. We also hypothesized that trajectory-based classification of AKI would be strongly linked to risk of death even after accounting for KDIGO severity stage.

## Methods

### Study design

We performed a secondary analysis of two independent, prospectively collected datasets (a single-center cohort and a multicenter sample) of patients admitted to the ICU. The single-center cohort (group 1; *n* = 1914) comprised patients with severe traumatic injury resulting in ICU admission between 2003 and 2005 [10]. The multicenter sample (group 2; *n* = 1867) was assembled as part of the identification of SNPs Predisposing to Altered ALI Risk (iSPAAR) study, a genome-wide case-control study of the risk for ARDS. The dataset included subjects at risk for ARDS enrolled from ICUs as part of the Molecular Epidemiology of Acute Respiratory Distress Syndrome (MEA) at the Massachusetts General Hospital [11] and subjects with ARDS enrolled by the National Heart, Lung, and Blood Institute (NHLBI) ARDS Network [12–15]. In both groups, patients with end-stage renal disease prior to admission were excluded. Of 2953 patients in group 1, 1012 were excluded because they were younger than 18 years of age, missing transfer data or receiving outpatient dialysis. Of 2236 patients in group 2, 369 were excluded because they were younger than 18 years of age, missing day 0 or day 1 creatinine values, and receiving outpatient dialysis. A flowchart of patients included in the study is provided in Additional file 1: Figure S1. The median number of SCr values was greater than 3.5.

### Data collection and measurement

AKI and stage of AKI were determined by changes in creatinine in accordance with the KDIGO criteria SCr thresholds for AKI during the first 72 h after study enrollment. We restricted our analysis to the first 72 h of ICU stay in an effort to focus on AKI due to the critical illness leading to ICU admission and to allow comparison with prior literature [16, 17]. We used SCr values obtained as part of clinical care, and the study did not mandate timing of measurement of SCr values. We did not include urine output in our determination of AKI, given the heterogeneity of urine output recordings and the high degree of missing data. We did not have access to preadmission baseline creatinine values for these subjects. Therefore, we set the baseline creatinine as the nadir observed in the first 72 h. AKI and severity were determined on the basis of maximal difference between the nadir creatinine and the maximal creatinine over this period.

We tested four definitions for the subphenotypes of resolving or nonresolving AKI. Patients were classified as resolving if they had a trajectory of SCr that was improving. All patients who did not meet the resolving criteria were classified as nonresolving. The SCr criteria for these definitions were based on a clinically meaningful change that reflected the magnitude of creatinine changes present in the KDIGO definition. We also explored variations of the definition for resolution that employed higher and lower changes in creatinine. We applied these definitions to group 1 and then compared hospital mortality for each set of resulting AKI subphenotypes (resolving/nonresolving) with that observed in patients without AKI. The definition demonstrating the largest separation in relative risk (RR) for death between resolving and nonresolving subphenotypes was carried forward to group 2.

Hospital mortality was defined as death prior to hospital discharge. Patients who died with less than two creatinine values were excluded. Hospital- and ICU-free days were defined as days free of the hospital and the ICU in the first 28 days after ICU admission. If a patient died within 28 days, then that patient had 0 ICU- or hospital-free days.

### Statistical analysis

We report continuous variables as means ± standard deviations and categorical variables as counts and percentages. Approximately 5% or less of the study participants were missing data on diabetes mellitus, cirrhosis, and vasopressors. For the regression analyses, data for participants with missing values for these covariates were imputed using chained equations and combined using Rubin rules [18]. No imputations were completed for exposure or outcome measures. Spaghetti plots were used to describe the longitudinal creatinine measurements and trajectories within each subphenotype designation.

RR regression was used to model the probability of death as a function of covariates using a generalized linear model with log-link and binomial error distribution [19]. In cases in which the model failed to converge with the binomial error (about 10% of the models), we substituted Gaussian error and used robust standard error estimates. The risk of death of each AKI subphenotype was compared with that of patients with no AKI. In group 1, we used unadjusted RR regression of the AKI subphenotype definitions and mortality. The definition that identified the greatest difference in risk of mortality between AKI subphenotypes was applied to group 2. In group 2, the first adjusted model included baseline age, sex, and race. Subsequent models added variables to the basic adjustment model that may confound or mediate the associations of interest. These variables were decided a priori on the basis of biological plausibility and prior literature [20–23]. The second model added body mass index, diabetes mellitus, Sepsis-related Organ Failure Assessment score (omitting creatinine), vasopressor use, sepsis (defined as two systemic inflammatory response syndrome criteria and a suspected source of infection), and the third model added maximum KDIGO stage of AKI during the first 72 h of hospital admission. Maximum KDIGO stage was included as an ordinal variable.

We conducted sensitivity analyses to evaluate whether associations between AKI subphenotype and mortality were robust within each KDIGO stage of AKI and used the likelihood ratio test to evaluate the statistical significance of the interaction. We used logistic regression incorporating an interaction term for the product of the maximum KDIGO stage and AKI subphenotype (i.e., no AKI, resolving and nonresolving). We also evaluated the association of timing of peak of SCr in the resolving subphenotype and mortality. Statistical analyses were conducted using Stata 13.0 software (StataCorp, College Station, TX, USA).

## Results

### Clinical data within groups 1 and 2

Baseline clinical data for patients in group 1 (trauma) and group 2 (mixed medical-surgical) are presented in Tables 1 and 2, respectively. Several differences between the two groups were noted. Group 1 tended to be younger and consisted of more patients transferred from another facility. Group 2 had more patients admitted with sepsis and requiring vasopressors early in their hospital stay. The populations in each group had a similar number of patients requiring mechanical ventilation. The trauma group had a mean Injury Severity Score of 23.6 and the medical-surgical mixed-sample group had a mean Acute Physiology and Chronic Health Evaluation III (APACHE III) score of 74. The percentage of patients with at least stage 1 AKI per KDIGO thresholds was 46% in group 1 and 69% in group 2. The proportion of patients with KDIGO stage 2 or 3 AKI among all patients with AKI was lower in group 1 (9%) than in group 2 (27%).

**Table 1:** Patient characteristics in group 1 (trauma)

Clinical variables	No acute kidney injury	Acute kidney injury	Total	AKI versus no AKI *p* value
Total	1037	877	1914	
Baseline demographics				
Age, years	36.8 ± 20.7	41.4 ± 19.9	38.9 ± 20.4	<0.01
Male sex, *n* (%)	678 (65)	691 (79)	1369 (72)	<0.01
Body mass index, kg/m^2^	25.6 ± 9.2	27.6 ± 6.8	26.5 ± 8	<0.01
Race, *n* (%)				
White	793 (77)	686 (78)	1479 (77)	0.96
Hispanic	91 (9)	55 (6)	146 (8)	
Black	64 (6)	56 (6)	120 (6)	
Other	65 (6)	60 (7)	125 (7)	
Unknown	24 (2)	20 (2)	44 (2)	
Comorbidities, *n* (%)				
Diabetes mellitus	34 (5)	61 (7)	95 (7.5)	0.01
Cerebrovascular disease	171 (17)	227 (26)	398 (21)	<0.01
Chronic kidney disease	4 (<1)	14 (2)	18 (1)	0.02
Injury Severity Score	23.1 ± 10.0	24.3 ± 10.2	23.6 ± 10.2	0.01
ICU events, *n* (%)				
Mechanical ventilation	701 (68)	657 (75)	1358 (71)	<0.01
Sepsis	235 (23)	236 (27)	471 (25)	0.01
Septic shock	30 (3)	70 (8)	100 (5)	<0.01
Vasopressors	89 (9)	133 (15)	222 (12)	<0.01
Admission status, *n* (%)				
Direct	719 (69)	645 (74)	1364 (71)	<0.01
Transfer	318 (31)	232 (26)	550 (29)	
Unknown	4 (<1)	1 (<1)	5 (<1)	
KDIGO stage of acute kidney injury, *n* (%)				
Stage 0	1037 (100)	0	1037 (54)	
Stage 1	0	807 (92)	807 (42)	
Stage 2	0	48 (5)	48 (3)	
Stage 3	0	22 (3)	22 (1)	

*AKI* Acute kidney injury, *ICU* Intensive care unit, *KDIGO* Kidney Disease: Improving Global Outcomes

Data shown as mean ± standard deviation, *n* (%) as appropriate

**Table 2:** Patient characteristics in group 2 (mixed medical-surgical)

Clinical variables	No acute kidney injury	Acute kidney injury	Total	AKI versus no AKI *p* value
Total	573	1294	1867	
Baseline demographics				
Age, years	56.8 ± 16.8	59.8 ± 18	58.9 ± 18	<0.01
Male sex, *n* (%)	246 (43)	543 (42)	794 (43)	0.78
Body mass index, kg/m^2^	27.5 ± 7.7	28.2 ± 7.5	27.9 ± 7.6	0.09
Race, *n* (%)				
White	573 (100)	1294 (100)	1867 (100)	1
Comorbidities, *n* (%)				
Diabetes mellitus	95 (17)	331 (26)	426 (23)	<0.01
SOFA score	7.0 ± 2.3	8.8 ± 2.9	8.3 ± 2.8	<0.01
SOFA without renal component	6.9 ± 2.2	7.7 ± 2.5	7.5 ± 2.5	<0.01
APACHE III score	64 ± 24.6	78 ± 26.8	74 ± 26.8	<0.01
ICU events, *n* (%)				
Mechanical ventilation	416 (78)	909 (70)	1325 (77)	<0.01
Sepsis	325 (56)	877 (68)	1202 (64)	<0.01
Vasopressors	134 (35)	571 (44)	705 (48)	<0.01
Diagnosis of ARDS	306 (53)	700 (37)	1006 (54)	<0.01
Clinical risk for ARDS, *n* (%)				
Pneumonia	298 (52)	702 (54)	1000 (54)	0.37
Sepsis	366 (64)	1006 (78)	1372 (73)	<0.01
Aspiration	74 (13)	158 (12)	232 (12)	0.75
Trauma	57 (10)	86 (7)	143 (8)	0.01
Other	23 (4)	31 (2)	54 (3)	0.06
Admission status, *n* (%)				
Direct	518 (90)	1192 (92)	1710 (92)	0.21
Transfer	55 (10)	102 (8)	157 (8)	
KDIGO stage of acute kidney injury, *n* (%)				
Stage 0	573 (100)	0	573 (31)	
Stage 1	0	944 (73)	944 (51)	
Stage 2	0	165 (13)	165 (9)	
Stage 3	0	185 (14)	185 (10)	

*Abbreviations*: *AKI* Acute kidney injury, *APACHE III* Acute Physiology and Chronic Health Evaluation III, *ARDS* Acute respiratory distress syndrome, *ICU* Intensive care unit, *KDIGO* Kidney Disease: Improving Global Outcomes, *SOFA* Sepsis-related Organ Failure Assessment

Data shown as mean ± standard deviation, *n* (%) as appropriate

### Identification of subphenotypes in group 1

**Fig. 1:**
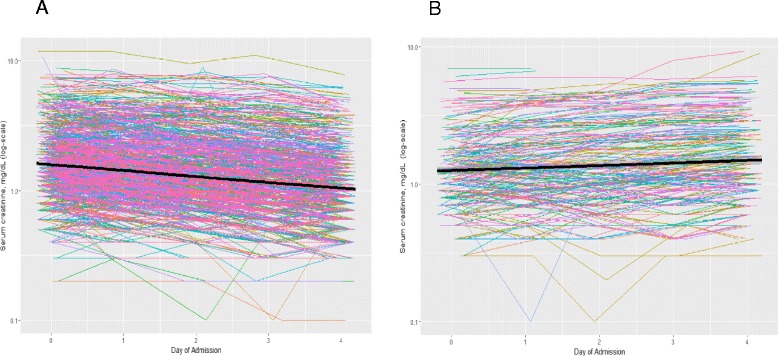
Diagram of the trajectory of patients with acute kidney injury (AKI) in the resolving and nonresolving subphenotype groups. Graphical representation of serum creatinine (SCr) trajectory in AKI subphenotypes. Spaghetti plots with trend lines of serial SCr values obtained over the first 72–96 h of admission in subjects exhibiting **a** resolving AKI subphenotype and **b** nonresolving AKI subphenotype

We tested four definitions of AKI subphenotypes in group 1 (trauma) and assessed the risk of death for each pair of subphenotypes relative to those without AKI (Additional file 1: Table S1). Definition 2 best separated the risk of death associated with the two AKI subphenotypes. The estimated increase in risk of death for the nonresolving subphenotype relative to patients without AKI was large (RR 2.95, 95% CI 2.08–4.19). In contrast, the resolving subphenotype had a markedly lower estimated effect on risk of death (RR 1.54, 95% CI 1.13–2.11) (Tables 3 and 4). Definition 2 criteria for the resolving subphenotype were a 0.3-mg/dl and/or 25% decrease in SCr from the maximum value during the first 72 h after study enrollment. Patients with AKI who did not meet these criteria for the resolving subphenotype were classified as having the nonresolving subphenotype. A graphical representation with a trend line of patients with resolving and nonresolving subphenotypes of AKI in group 1 is shown in Fig. 1. This graph shows the consistency in creatinine trajectories within the resolving and nonresolving AKI subphenotypes. Versions of Additional file 1: Tables S1 and S2 including the AKI subphenotypes are provided in Additional file 1: Tables S2–S4.

**Table 3:** Clinical outcomes in group 1 (trauma)

		AKI Severity			AKI Subphenotype	
Outcomes	No AKI	Stage 1	Stage 2	Stage 3	Resolving	Nonresolving
Number of patients	1037	807	48	22	647	230
Mortality, *n* (%)	72 (7)	102 (12)	8 (16)	6 (27)	72 (11)	42 (20)
Hospital-free days, 28 days, *n* (IQR)	16 (5–22)	11 (0–19)	8 (0–16.5)	0.5 (0–12)	11 (0–18)	4 (0–18)
ICU-free days, 28 days, *n* (IQR)	24 (17–26)	21 (7–25)	16.5 (0–24)	11.5 (0–23)	21 (9–24)	15 (0–25)

*AKI* Acute kidney injury, *ICU* Intensive care unit

Data are presented as count (percent) or median (interquartile range [IQR]), unless otherwise indicated

**Table 4:** Clinical outcomes in group 2 (mixed medical-surgical)

		AKI severity			AKI subphenotype	
Outcomes	No AKI	Stage 1	Stage 2	Stage 3	Resolving	Nonresolving
Number of patients	573	944	165	185	875	419
Mortality, *n* (%)	62 (11)	148 (16)	35 (21)	39 (21)	115 (13)	107 (26)
Hospital-free days, 28 days, *n* (IQR)	19 (11–24)	17 (7–23)	16 (1–22)	13 (0–20)	17 (8–23)	14 (0–21)
ICU-free days, 28 days, *n* (IQR)	21 (12–25)	20 (11–24)	18 (4–24.5)	18 (0–24)	21 (13–25)	17 (0–23)

*AKI* Acute kidney injury, *ICU* Intensive care unit

Data are presented as count (percent) or median (interquartile range [IQR]), unless otherwise indicated

### AKI subphenotypes and risk of mortality in group 2

We applied the subphenotype criteria in definition 2 to subjects in group 2. Similar to our findings in group 1, we found that mortality was higher for patients with a nonresolving AKI subphenotype (26%) than for those with a resolving AKI subphenotype (13%), and mortality for both was numerically higher than that observed for subjects without AKI (11%). The subjects with the nonresolving subphenotype experienced fewer hospital and ICU-free days than those with the resolving subphenotype (Tables 3 and 4). Increasing severity of AKI was also associated with higher mortality and fewer hospital- and ICU-free days in group 2. The mean ± standard deviation of APACHE III scores in the resolving AKI group was 78 ± 26, and in the nonresolving AKI group it was 78.9 ± 28. The rates of mechanical ventilation and vasopressor use were similar between the AKI subphenotypes.

Using multivariable logistic regression, we built models in a stepwise fashion to evaluate the association of trajectory with hospital mortality (Table 5). Model 2 included patient demographics, comorbidities, and ICU factors. In model 2, a nonresolving subphenotype of AKI was associated with a 50% increase in RR for in-hospital mortality compared with patients without AKI (RR 1.52, 95% CI 1.13–2.05). A resolving subphenotype compared with no AKI was not associated with mortality (RR 0.86, 95% CI 0.63–1.17). Next, we tested whether the association between AKI subphenotypes and mortality was independent of maximal AKI stage. We added AKI stage to the multivariable model (model 3) and found that patients with a nonresolving subphenotype of AKI still had a 68% higher risk of death (RR 1.68, 95% CI 1.15–2.44).

**Table 5:** Adjusted relative risk for hospital mortality by trajectory and KDIGO classification in group 2 (mixed medical-surgical)

			Relative risk (95% CI)		
	Participants	Events, *n* (%)	Model 1	Model 2	Model 3
No AKI	573	62 (10.8)	1.00 (reference)	1.00 (reference)	
KDIGO AKI stage					
Stage 1	944	148 (15.7)	1.27 (0.96–1.69)	1.05 (0.78–1.43)	–
Stage 2	165	35 (21.2)	1.75 (1.19–2.57)	1.23 (0.85–1.78)	–
Stage 3	185	39 (21.1)	1.68 (1.14–2.48)	1.2 (0.84–1.73)	–
Subphenotype					
Resolving	875	115 (13.1)	1.05 (0.78–1.43)	0.86 (0.63–1.17)	0.95 (0.64–1.41)
Nonresolving	419	107 (25.5)	2.04 (1.52–2.75)	1.52 (1.13–2.05)	1.68 (1.15–2.44)

*AKI* Acute kidney injury, *KDIGO* Kidney Disease: Improving Global Outcomes

Model 1: Age, sex, race

Model 2: Body mass index, diabetes mellitus, Sepsis-related Organ Failure Assessment score, vasopressor use, sepsis

Model 3: Maximum KDIGO stage of AKI

### Risk of mortality by AKI subphenotype within KDIGO stages in group 2

We found that, within every KDIGO AKI stage, the subjects with the nonresolving subphenotype had higher mortality than those with the resolving subphenotype (*p* for interaction = 0.035) (Table 6). For example, among subjects with KDIGO stage 1 AKI, those with the nonresolving subphenotype had more than twice the risk of death (RR 2.06, 95% CI 1.52–2.80) compared with a resolving subphenotype.

**Table 6:** Adjusted relative risk for in-hospital mortality within KDIGO acute kidney injury stage by trajectory of acute kidney injury within group 2

KDIGO AKI stage	AKI Subphenotype	*n* (%)	Deaths, *n* (%)	RR^a^ (95% CI)
No AKI	–	573 (31)	62 (10.8)	
Stage 1	Resolving	610 (33)	70 (11.5)	1.0 (reference)
	Nonresolving	334 (17)	78 (23.4)	2.06 (1.52–2.80)
Stage 2	Resolving	131 (7)	21 (16.0)	1.0 (reference)
	Nonresolving	34 (2)	14 (41.2)	2.41 (1.36–4.29)
Stage 3	Resolving	134 (7)	24 (17.9)	1.0 (reference)
	Nonresolving	51 (3)	15 (29.4)	1.59 (0.87–2.92)

*AKI* Acute kidney injury, *KDIGO* Kidney Disease: Improving Global Outcomes, *RR* Relative risk

*p* for interaction between KDIGO stage and trajectory on risk of death: *p* = 0.035

^a^ Adjusted for age, sex, race (all white), body mass index, diabetes mellitus, Sepsis-related Organ Failure Assessment without renal component, vasopressors, sepsis

### Timing of SCr peak in the resolving AKI subphenotype and risk of mortality

We completed additional sensitivity analyses to evaluate, in the subgroup of patients with a resolving AKI subphenotype, if the day of peak in SCr influenced the association with mortality. The total number of patients in group 2 with a resolving AKI subphenotype was 914. Of these 914 patients, 517 had an SCr peak on day 0, 239 peaked on day 1, 117 peaked on day 2, and 41 peaked on day 3. The mortality ranged from 11% if the SCr peaked on day 0 to 14% if the peak was on day 2 or on day 3 (Additional file 1: Table S4).

## Discussion

In two distinct, large, heterogeneous ICU populations, we demonstrated that the trajectory of SCr defines subphenotypes of AKI and that these subphenotypes are independently associated with hospital mortality, length of hospital stay, and length of ICU stay. Despite significant differences in baseline clinical characteristics, etiologies for renal dysfunction, and ICU-level therapies between group 1 (trauma) and group 2 (mixed medical-surgical), the association between AKI subphenotypes and short-term clinical outcomes persisted. Critically ill patients with a nonresolving subphenotype compared with no renal dysfunction had a greater than 60% increased risk of hospital mortality. Additionally, patients with a resolving subphenotype had the same risk of death as those having no AKI. Of even greater interest, when we controlled for KDIGO severity of AKI, both AKI subphenotypes maintained their associations with hospital mortality. Notably, even among patients with KDIGO stage 1 AKI, the nonresolving subphenotype was associated with double the risk of death compared with the resolving subphenotype. These findings show that there exists considerable variability in risk for poor outcomes within the KDIGO stages of AKI and that even relatively small decreases in SCr from the maximal value have important implications for hospital outcomes.

Our findings extend and clarify those of prior studies seeking to subclassify patients with AKI. In previous studies, researchers evaluated the relationship between duration of AKI (transient versus persistent) and hospital mortality but found contradictory results [16, 17, 24]. In two studies, researchers found that separating patients on the basis of duration of AKI between transient (less than 72 h) and persistent (greater than 72 h) did not lead to the identification of patients at increased risk for mortality [16, 17]. In contrast, researchers in a third study of patients experiencing AKI after elective surgery found that a group of patients with a long duration of AKI were at higher risk of death than a group with a short AKI duration [25]. One potential explanation for these conflicting results is that the authors mandated that the baseline creatinine be based on an outpatient value, which is often unavailable in ICU patients [9]. For baseline values that were missing, researchers in these studies used mathematical formulas to impute this baseline SCr value. Given that these formulas were derived from relatively healthy outpatients in the steady state [26] with a “normal” expected glomerular filtrate rate, the application of these formulas to critically ill patients may lead to significant inaccuracies [27, 28]. In our present study, we sought to address this problem by developing an approach to subclassify AKI on the basis of patterns of SCr values after ICU admission, obviating the need for an outpatient or premorbid SCr value.

Our approach shows robust associations between nonresolving AKI and poor hospital outcomes in two large ICU cohorts. We selected the definition used to identify the two AKI subphenotypes—resolving and nonresolving—in a cohort of patients with a low prevalence of preexisting kidney disease and in whom the temporal relationship between injury and development of AKI was known. We then applied this definition for AKI subphenotypes to a considerably more diverse ICU population (mixed medical-surgical population). Markers of severity of illness, such as APACHE III score, vasopressor use, and mechanical ventilation, did not differentiate the AKI subphenotypes on ICU admission. Thus, we have identified a novel marker for risk of death over and above traditional risk factors for AKI and even well-established AKI severity scores. Of interest, three of the four AKI subphenotype definitions tested in group 1 showed differential risks for mortality. This suggests that the optimal definition may remain undetermined. Nonetheless, our work clearly shows that patients with a rapidly improving SCr have a very different outcome from a nonresolving SCr.

There are several ways in which classifying patients by AKI subphenotypes could be useful. First, refining the AKI phenotype could aid studies evaluating the pathophysiology of AKI by providing a more uniform study population. For instance, identifying AKI subphenotypes might enrich genetic studies for a particular pathologic subtype of AKI, thus reducing misclassification and improving the power to identify genetic risk factors associated with the development of AKI. Second, knowledge of AKI subphenotypes that are associated with differential risk of clinical outcomes could aid in triage decisions for severely ill patients. Third, enrollment in clinical trials could be directed at subgroups of patients most at risk of poor outcomes who might benefit from a novel therapeutic intervention. Fourth, existing biomarkers, such as neutrophil gelatinase-associated lipocalin, have had mixed results in identifying patients with AKI. In the cohorts studied, the KDIGO stage of AKI influenced the effectiveness of biomarkers to predict the development of AKI with lower test performance characteristics in patients with less severe AKI [29–31]. Because the trajectory of SCr identifies patients with increased risk of poor clinical outcomes, it is possible that identifying AKI subphenotypes may improve biomarker performance. Grouping patients with a resolving versus nonresolving trajectory increases the heterogeneity of AKI and likely limits biomarker development.

Our study has some limitations. First, we did not have data on urine output, which can define an AKI event by KDIGO criteria. The inclusion of urine output may have increased the number of subjects classified as AKI cases and improved our ability to categorize AKI [32]. However, given that our definitions for the subphenotypes were based on the trajectory of SCr values, a better marker of true glomerular filtration rate and risk of death [26, 33], it is unlikely that our findings would have been different to a meaningful degree. Second, to determine severity of AKI, we used the nadir of SCr rather than a preadmission baseline value. Prehospital SCr values are often lacking in ICU patients, particularly during the early part of their admission, and thus an approach that bypasses the requirement for this information could allow for a more timely identification of AKI subphenotypes. Prehospital SCr values may have improved the accuracy of the KDIGO severity of AKI, but it is unlikely to have influenced the association of trajectory with outcomes. Additionally, if we had used prehospital SCr, then fluid administration in the emergency room or ICU may have created a dilution effect and decreased the incidence of AKI. In contrast, using a nadir of SCr overcomes this limitation by accounting for changes in SCr secondary to fluid administration. Third, group 1 included few patients with KDIGO AKI stage 2 or 3. The lack of these stages of AKI may limit the generalizability of trajectory-based findings to a trauma population with severe AKI. Fourth, previous studies have compared patients with and without AKI and have found an association with fluid accumulation and mortality in AKI [34, 35]. While most patients in the study were likely vigorously hydrated early in their ICU care, in accordance with ICU practice, we lacked accurate fluid balance data. Thus, it is unknown how fluid balance was associated with AKI subphenotype during a patient’s hospital stay.

Our study has several strengths. First, our definitions of AKI subphenotypes are based on changes in serial SCr values that are widely available in many existing ICU datasets. This will allow our subphenotype definitions to be quickly determined in large numbers of critically ill patients and their relationships with outcomes to be assessed. Furthermore, future application of our definitions to prospectively identify AKI subphenotypes should be straightforward. Second, we observed large and robust increases in risk of death associated with the nonresolving AKI subphenotype that were independent of the most widely established measure of AKI severity, the KDIGO staging system. This suggests that our approach could add value to the current classification schemes for AKI. Third, we used a large and diverse set of ICU patients who ranged from victims of major trauma to patients with severe pneumonia enrolled in randomized clinical trials of ARDS. Furthermore, the patients in group 1 were racially diverse. These factors suggest that our findings will be generalizable to other critically ill patient populations. Our results need to be validated in additional larger multicenter cohorts of critically ill patients.

## Conclusions

We identified two distinct subphenotypes of AKI that are associated with different risks for hospital mortality. These subphenotypes also identify subjects with differences in risk for other short-term outcomes, including prolonged course in the ICU and time requiring mechanical ventilator support. Our observations should prompt future research to molecularly characterize these subphenotypes and to evaluate predictive biomarkers that might enable earlier identification of AKI subphenotypes. Refining the classification of AKI in ICU populations will allow recognition of patients at high risk of poor outcomes who might be targeted for enrollment in clinical trials, improve triage decisions, and focus the search for genetic risk factors.

## Additional file

Additional file 1: Table S1. Definitions of AKI subphenotypes and unadjusted relative risk of trajectory with hospital mortality in group 1 (trauma). **Table S2.** Patient characteristics in group 1 (trauma) by AKI subphenotypes. **Table S3.** Patient characteristics in group 2 (mixed medical and surgical) by AKI subphenotypes. **Table S4.** Patient characteristics with a resolving AKI subphenotype in group 2. **Figure S1.** Flowchart of patient inclusion. (DOCX 46 kb)

## Abbreviations

AKI: Acute kidney injury
ALTA: Drug Study of Albuterol to Treat Acute Lung Injury
APACHE III: Acute Physiology and Chronic Health Evaluation III
ARDS: Acute respiratory distress syndrome
COPD: Chronic obstructive pulmonary disease
EDEN: Early Versus Delayed Enteral Feeding to Treat People With Acute Lung Injury or Acute Respiratory Distress Syndrome
EDEN-OMEGA: Early Versus Delayed Enteral Feeding and Omega-3 Fatty Acid/Antioxidant Supplementation for Treating People With Acute Lung Injury or Acute Respiratory Distress Syndrome
FACTT: Fluids and Catheters Treatment Trial
ICU: Intensive care unit
iSPAAR: Identification of SNPs Predisposing to Altered ALI Risk
KDIGO: Kidney Disease: Improving Global Outcomes
MEA: Molecular Epidemiology of Acute Respiratory Distress Syndrome
RR: Relative risk
SCr: Serum creatinine
SOFA: Sepsis-related Organ Failure Assessment
