# Supplementary material for: Acute kidney injury subphenotypes based on creatinine trajectory identifies patients at increased risk of death
Source: Crit Care. 2016 Nov 17;20:372. doi: 10.1186/s13054-016-1546-4 (PMC5112626; doi:10.1186/s13054-016-1546-4)

**Additional file 1**

**Table S1. Definitions of AKI Sub-phenotypes and Unadjusted Relative Risk of Trajectory with Hospital Mortality in Group 1 –Trauma**

| **AKI Sub-phenotype** | **Trajectory of SCr Criteria** | **Dead n (%)** | **Alive n (%)** | **RR (95% CI)** |
| --- | --- | --- | --- | --- |
|  | Total | 186 (10) | 1728 (90) |  |
|  | **Definition 1** |  |  |  |
| No AKI | <0.3 mg/dl change in SCr from creatinine nadir | 72 (7) | 965 (93) | 1.0* |
| Resolving AKI | 0.3 mg/dl and/or 50% decrease in SCr from maximum in the first 72 hours | 72 (11) | 596 (89) | 1.55 (1.14, 2.12) |
| Non-resolving AKI | AKI patients not meeting the definition of resolving | 42 (20) | 167 (80) | 2.89 (2.04, 4.11) |
|  | **Definition 2** |  | |  |
| No AKI | <0.3 mg/dl change in SCr from creatinine nadir | 72 (7) | 965 (93) | 1.0* |
| Resolving AKI | 0.3 mg/dl and/or 25% decrease in SCr from maximum in the first 72 hours | 72 (11) | 600 (89) | 1.54 (1.13, 2.11) |
| Non-resolving AKI | AKI patients not meeting the definition of resolving | 42 (20) | 163 (80) | 2.95 (2.08, 4.19) |
|  | **Definition 3** |  |  | |
| No AKI | <0.3 mg/dl change in SCr from creatinine nadir | 72 (7) | 965 (93) | 1.0* |
| Resolving AKI | 0.6 mg/dl and/or 25% decrease in SCr from maximum in the first 72 hours | 49 (10) | 466 (90) | 1.37 (.97, 1.94) |
| Non-resolving AKI | AKI patients not meeting the definition of resolving | 65 (18) | 297 (82) | 2.59 (1.89, 3.54) |
|  | **Definition 4** |  | |  |
| No AKI | <0.3 mg/dl change in SCr from creatinine nadir | 72 (7) | 965 (93) | 1.0 * |
| Resolving AKI | 0.6 mg/dl and/or 50% decrease in SCr from maximum in the first 72 hours | 17 (13) | 114 (87) | 1.87 (1.14, 3.07) |
| Non-resolving AKI | AKI patients not meeting the definition of resolving | 97 (13) | 649 (87) | 1.87 (1.40, 2.50) |

* No AKI used as the reference

**Table S2. Patient Characteristics in Group 1 (Trauma) by AKI Sub-phenotypes**

| **Clinical Variable** | **Group 1** | | |  |
| --- | --- | --- | --- | --- |
|  | **No AKI** | **AKI** | | **Total** |
|  |  | **Resolving AKI** | **Non-Resolving AKI** |  |
| **Total** | 1037 | 647 | 230 | 1914 |
| **Baseline Demographics** |  |  |  |  |
| Age (year) | 36.8 +/- 20.7 | 40.2 +/- 19.5 | 44.9 +/-20.7 | 38.9 +/- 20.4 |
| Male (%) | 678 (65) | 502 (78) | 189 (82) | 1369 (72) |
| Body Mass Index (kg/m^2^) | 25.6 +/- 9.2 | 27.2 +/- 6.5 | 28.5 +/-7.6 | 26.5 +/- 8 |
| Race (%) |  |  |  |  |
| Caucasian | 793 (77) | 509 (79) | 177 (8) | 1479 (77) |
| Hispanic | 91 (9) | 43 (7) | 12 (5) | 146 (8) |
| Black | 64 (6) | 36 (6) | 20 (9) | 120 (6) |
| Other | 65 (6) | 45 (7) | 15 (7) | 125 (7) |
| Unknown | 24 (2) | 14 (2) | 6 (3) | 44 (2) |
| **Injury Severity Score** | 23.1 +/- 10 | 24.5 +/- 10 | 23.8 +/- 10.8 | 23.6 +/- 10.2 |
| **Co-morbidities (%)** |  |  |  |  |
| Diabetes Mellitus* | 34 (5) | 36 (8) | 25 (15) | 95 (7.5) |
| Cerebrovascular Disease | 171 (17) | 156 (24) | 71 (31) | 398 (21) |
| Chronic Kidney Disease | 4 (<1) | 7 (1) | 7 (3) | 18 (<1) |
| **ICU Events** |  |  |  |  |
| Mechanical Ventilation | 701 (68) | 493 (76) | 164 (71) | 1358 (71) |
| Sepsis | 235 (23) | 180 (28) | 56 (24) | 471 (25) |
| Septic Shock | 30 (3) | 38 (6) | 32 (14) | 100 (5) |
| Vasopressors | 89 (9) | 76 (12) | 57 (25) | 222 (12) |
| **Admission Status** |  |  |  |  |
| Direct | 715 (69) | 477 (74) | 166 (73) | 1359 (71) |
| Transfer | 318 (31) | 170 (26) | 62 (27) | 550 (29) |
| Unknown | 4 | 0 | 1 | 5 |
| **KDIGO Stage of AKI** |  |  |  |  |
| Stage 0 | 1037 (100) | 0 | 0 | 1037 (54) |
| Stage 1 | 0 | 596 (92) | 211 (92) | 807 (42) |
| Stage 2 | 0 | 39 (6) | 9 (4) | 48 (3) |
| Stage 3 | 0 | 12 (2) | 10 (4) | 22 (1) |
| Total | 1037 | 640 | 230 | 1914 |

Data shown as mean+/- standard deviation, n(%) as appropriate

SOFA – Sepsis related Organ Failure Assessment

**Table S3. Patient Characteristics in Group 2 (Mixed Medical and Surgical) by AKI Sub-phenotypes**

| **Clinical Variables** | **Group 2** | | |  |
| --- | --- | --- | --- | --- |
|  | **No AKI** | **AKI** | | **Total** |
|  |  | **Resolving AKI** | **Non-Resolving AKI** |  |
| **Total** | 573 | 875 | 419 | 1867 |
| **Baseline Demographics** |  |  |  |  |
| Age (year) | 56.8 +/-16.8 | 59.5 +/- 18 | 60.4 +/- 18 | 58.9 +/- 18 |
| Male | 246 (43) | 359 (41) | 184 (44) | 794 (43) |
| Body Mass Index (kg/m^2^) | 27.5 +/- 7.7 | 28.3 +/- 7.4 | 27.8 +/-7.7 | 27.9 +/- 7.6 |
| Race (%) |  |  |  |  |
| Caucasian | 573 (100) | 875 (100) | 419 (100) | 1867 (100) |
| **Co-Morbidities (%)** |  |  |  |  |
| Diabetes Mellitus* | 95 (17) | 223 (26) | 108 (26) | 426 (23) |
| SOFA Score | 7.0 +/-2.3 | 8.9 +/-2.9 | 8.6 +/-2.8 | 8.3 +/- 2.8 |
| SOFA Score without renal | 6.9 +/- 2.2 | 7.7 +/-2.5 | 7.7 +/- 2.5 | 7.5 +/ 2.5 |
| APACHE III | 64 +/- 24.6 | 78 +/- 26 | 79 +/-28 | 74 +/-26.8 |
| **ICU Events** |  |  |  |  |
| Mechanical Ventilation± | 416 (78) | 599 (74) | 310 (79) | 1325 (77) |
| Sepsis | 325 (56) | 628 (72) | 249 (59) | 1202 (64) |
| Vasopressors~ | 134 (35) | 382 (52) | 189 (55) | 706 (48) |
| **Diagnosis of ARDS** | 306 (53) | 445 (51) | 255 (61) | 1006 (54) |
| **Clinical Risk for ARDS** |  |  |  |  |
| Pneumonia | 298 (52) | 469 (54) | 233 (56) | 1000 (54) |
| Sepsis | 366 (64) | 698 (80) | 308 (74) | 1372 (73) |
| Aspiration | 74 (13) | 103 (12) | 55 (13) | 232 (12) |
| Trauma | 57 (10) | 61 (7) | 25 (6) | 143 (8) |
| Other | 23 (4) | 16 (2) | 15 (4) | 54 (3) |
| **Admission Status** |  |  |  |  |
| Direct | 518 (90) | 813 (93) | 379 (90) | 1710 (92) |
| Transfer | 55 (10) | 62 (7) | 40 (10) | 157 (8) |
| **KDIGO Stage of AKI** |  |  |  |  |
| Stage 0 | 573 (100) | 0 | 0 | 573 (31) |
| Stage 1 | 0 | 610 (70) | 334 (80) | 944 (51) |
| Stage 2 | 0 | 131 (15) | 34 (8) | 165 (9) |
| Stage 3 | 0 | 134 (15) | 51 (12) | 185 (10) |

Data shown as mean+/- standard deviation, n(%) as appropriate

SOFA – Sepsis related Organ Failure Assessment

APACHE III– Acute Physiology and Chronic Health Evaluation

* 1848 with information on diabetes mellitus

±1765 with information on mechanical ventilation

~ 1468 with information on vasopressors

**Table S4. Patient Characteristics with a Resolving AKI Sub-phenotype in Group 2**

| **Peak Serum Creatinine (days)** | **Number of Patients** | **Mortality (%)** |
| --- | --- | --- |
| 0 | 517 | 11 |
| 1 | 239 | 13 |
| 2 | 117 | 14 |
| 3 | 41 | 14 |
| Total | 914 | 13 |

**Figure S1 – Flow Chart of Patient Inclusion**


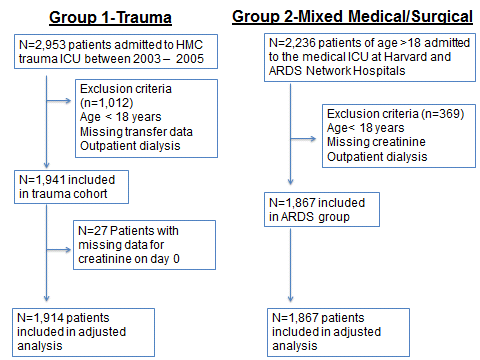

Supplement: Additional file 1: Table S1. — Definitions of AKI subphenotypes and unadjusted relative risk of trajectory with hospital mortality in group 1 (trauma). Table S2. Patient characteristics in group 1 (trauma) by AKI subphenotypes. Table S3. Patient characteristics in group 2 (mixed medical and surgical) by AKI subphenotypes. Table S4. Patient characteristics with a resolving AKI subphenotype in group 2. Figure S1. Flowchart of patient inclusion. (DOCX 46 kb) [file 13054_2016_1546_MOESM1_ESM.docx]
